# SNP Detection from *De Novo* Transcriptome Sequencing in the Bivalve *Macoma balthica*: Marker Development for Evolutionary Studies

**DOI:** 10.1371/journal.pone.0052302

**Published:** 2012-12-26

**Authors:** Eric Pante, Audrey Rohfritsch, Vanessa Becquet, Khalid Belkhir, Nicolas Bierne, Pascale Garcia

**Affiliations:** 1 Littoral, Environnement et Sociétés Joint Research Unit 7266 Centre national de la recherche scientifique, Université de La Rochelle, La Rochelle, France; 2 Joint Research Unit 5554, Institut des Sciences de l’Entreprise et du Management, Université Montpellier II, Montpellier, France; 3 Joint Research Unit 5554â, Institut des Sciences de l’Entreprise et du Management, Université Montpellier II, Station Méditerranéenne de l’Environnement Littoral, Montpellier, France; Radboud University Medical Centre, NCMLS, The Netherlands

## Abstract

Hybrid zones are noteworthy systems for the study of environmental adaptation to fast-changing environments, as they constitute reservoirs of polymorphism and are key to the maintenance of biodiversity. They can move in relation to climate fluctuations, as temperature can affect both selection and migration, or remain trapped by environmental and physical barriers. There is therefore a very strong incentive to study the dynamics of hybrid zones subjected to climate variations. The infaunal bivalve *Macoma balthica* emerges as a noteworthy model species, as divergent lineages hybridize, and its native NE Atlantic range is currently contracting to the North. To investigate the dynamics and functioning of hybrid zones in *M. balthica*, we developed new molecular markers by sequencing the collective transcriptome of 30 individuals. Ten individuals were pooled for each of the three populations sampled at the margins of two hybrid zones. A single 454 run generated 277 Mb from which 17K SNPs were detected. SNP density averaged 1 polymorphic site every 14 to 19 bases, for mitochondrial and nuclear loci, respectively. An 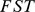 scan detected high genetic divergence among several hundred SNPs, some of them involved in energetic metabolism, cellular respiration and physiological stress. The high population differentiation, recorded for nuclear-encoded ATP synthase and NADH dehydrogenase as well as most mitochondrial loci, suggests cytonuclear genetic incompatibilities. Results from this study will help pave the way to a high-resolution study of hybrid zone dynamics in *M. balthica*, and the relative importance of endogenous and exogenous barriers to gene flow in this system.

## Introduction

Understanding the adaptation of organisms to their environment is becoming a pressing matter, in the face of today’s anthropogenic pressures and rapid climate change (e.g. [1]). In response to elevated temperatures, the range of many terrestrial and marine species has shifted toward cooler zones (higher latitudes, altitudes, or deeper waters), leading to profound modifications in biogeographic, ecological, and evolutionary patterns [2]–[4].

In this context, hybrid zones are of particular interest, as they are an important component of both animal and plant systems, represent a key process in the maintenance of biodiversity, and sit at the very core of the process of speciation (e.g. [5] and the FroSpects workshop on hybridization and speciation 2012). Because climate can affect both selection (by the differential survival of cold-adapted and heat-adapted genotypes) and connectivity (by influencing, for example, the number of migrants produced), hybrid zones can move in response to climate change (e.g. [6], [7]). On the other hand, hybrid zones are expected to be efficiently trapped by exogenous factors such as natural barriers to gene flow [8] or environmental boundaries unrelated to climate. There is therefore a very strong incentive to study the mechanisms involved in the dynamics of hybrid zones subjected to climate change.

*Macoma balthica*, an infaunal tellinid bivalve from marine and estuarine soft-bottom habitats of the northern hemisphere, is a particularly well suited model system to study the response of marine hybrid zones to changing environmental conditions. The natural range of *M. balthica* is currently contracting poleward [9], [10], in parallel with increasing sea surface temperatures in the Bay of Biscay (NE Atlantic; [11]). Previously found along the Atlantic side of Spain, the southern species boundary has shifted over 600 km north, to the Gironde Estuary (France) during the last 40 years. As *M. balthica* is a major element in the diet of several migratory bird, macro-invertebrates and fish [12]–[15], its disappearance from European coasts may lead to profound ecosystemic alterations. A putative hybrid zone was recently detected among southern European populations of *M. balthica* [16], raising the question of the evolutionary equilibrium of meridional *M. balthica* populations. Whether this zone is moving northward in response to climate change is unclear, as it could be trapped by natural barriers to gene flow such as a the Brittany peninsula (e.g. [17], [18]). Also, the northern lineage of *M. balthica* could benefit from warm-adapted alleles from the southern lineage through introgressive hybridization.

Next-generation sequencing technologies have recently catalyzed genome-wide studies of population differentiation, local adaptation and hybridization on non-model organisms (reviewed in [19]–[21]). In particular, transcriptome-wide sequencing was shown to be an effective way to obtain a large number of co-dominant genetic markers, even when no reference genome is available (e.g. [22]–[25]).

In this communication, we present results of a transcriptome-wide scan for single nucleotide polymorphisms (SNPs) with the 454 technology [26], performed in preparation for a large-scale study of the maintenance and dynamics of hybrid zones in the context of global climate change. This preliminary scan for genetic markers of adaptive differentiation (1) provides data on the polymorphism of *M. balthica*, (2) outlines loci putatively affected by selection, and (3) offers ground for discussing cytonuclear disequilibrium among *M. balthica* populations.

## Results

### Assembly Statistics

**Figure 1:**
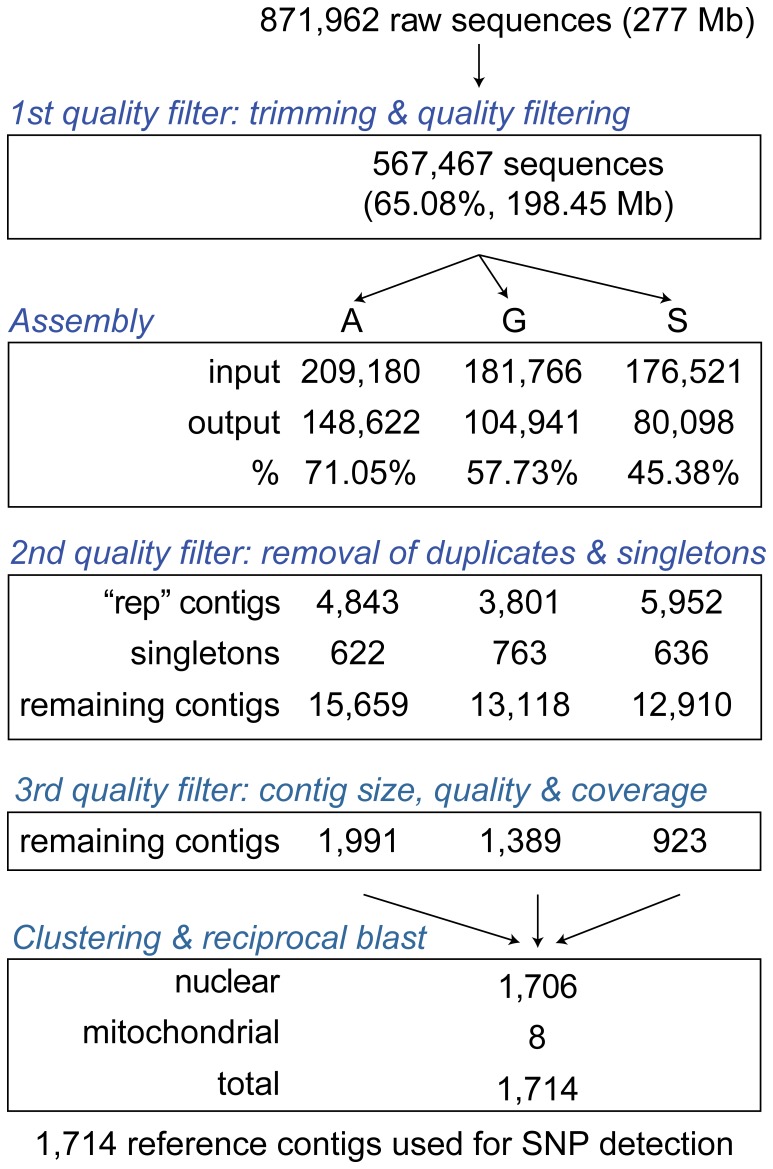
Summary of assembly, trimming and quality filtering of raw 454 sequence data.

**Figure 2:**
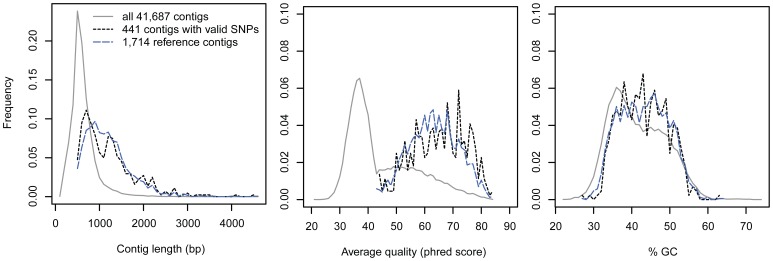
Effect of data quality filtering on contig length, average quality, and GC content. Solid line: all contigs after removal of singletons and repetitive regions; black dashed line: contigs with high-quality SNPs; blue dashed line: reference contigs.

A total of 871,962 sequences were obtained with one 454 GS FLX Titanium run, representing 277 Mbases. Only 4,303 contigs (1,991, 1,389 and 923 for Aytré, Gdansk and Somme respectively) were retained after assembly and removal of singletons and repetitive regions (Figure 1). Contig length ranged from 43 bp to 4,541 bp with a median of 513 bp. Distribution of average qualities was right-skewed with a peak score at 37. GC content varies from 21.3% to 73.8% with a median of 35.4%. Quality filtering based on contigs size (≥400 bp) and base call quality (≥42) significantly improved average contig quality and stabilized GC content (Figure 2). After the step of clustering and reciprocal blast, the remaining 1,714 contigs (1.96 Mbases) were used as reference genome for mapping and SNP calling. Medians of contig length, average quality and GC content were 1,061 bp, 63 and 42.8%, respectively.

### Contig Identification and Functional Annotation

**Figure 3:**
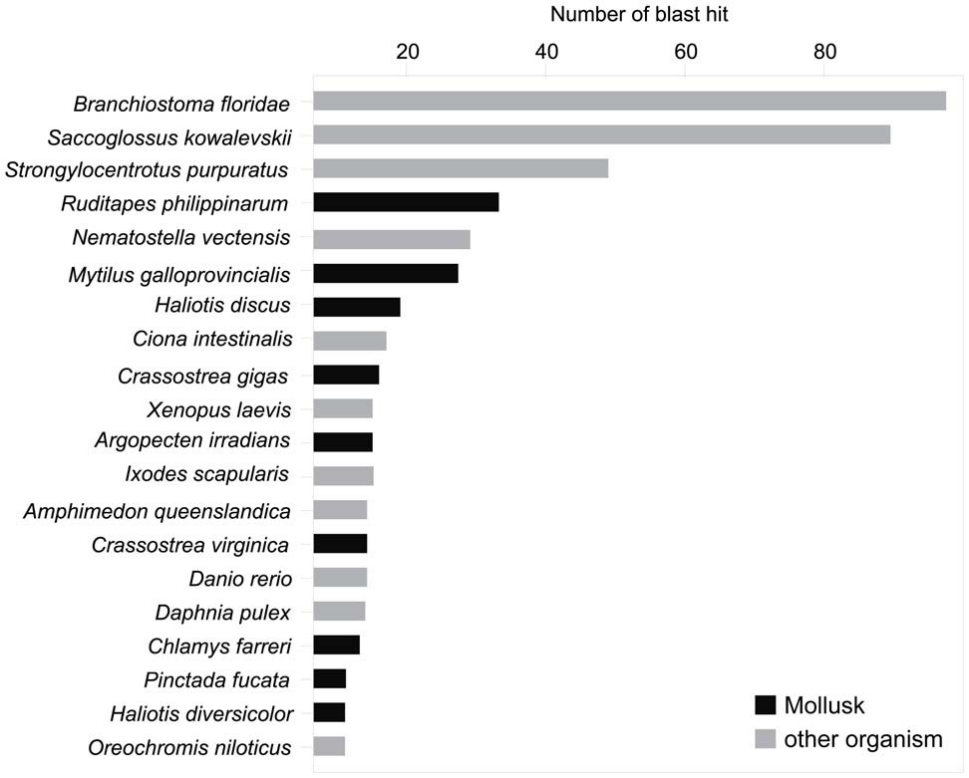
Species identification of the best blast searches. Results shown for the first 20 species identified, based on the 1,714 reference contigs.

Of 1,714 reference contigs, 856 returned a blast hit, and, from these, 512 were associated to a Gene Ontology term. The species that returned the most of top blast hits was *Branchiostoma floridae*, a cephalochordate of the Branchiostomidae family. Among the top ten best-hit species, four mollusks were detected, including three bivalves (*Ruditapes*, *Mytilus*, and *Crassostrea*; Figure 3). A more thorough characterization of the transcriptome is ongoing. Eight contigs (cumulative size of 10,467 bp) were retained as sequences of mitochondrial origin, matching the following genes: *cytb*, *nad2*, *nad4*, *nad5*, *cox1*, *cox3* and 16S (Sanger-sequencing validation of the homology of these contigs for mitochondrial genes is ongoing).

Only sequences from the mitochondrial *cox1* and *cox3* genes are currently available for *M. balthica* on GenBank. Reads mapped on two contigs (A_c14306 and G_c395, corresponding to *cox1* and *cox3*, respectively) were used to build three consensus sequences corresponding to the three sampled populations. For *cox1*, consensuses were close to known haplotypes published in Luttikhuizen *et al*. [27], [28] and Nikula *et al*. [29]. The consensus from the Aytré and Somme populations closely matched Nikula’s haplotype 37 (*M. balthica rubra* b1 clade; GenBank accession n. EF044130) and Luttikhuizen’s haplotype e (AF443220) [27], while sequences from the Gdansk population closely matched Luttikhuizen’s haplotype O (AY62262) from the Baltic Sea [28]. Similarly, for *cox3*, all three consensus sequences exactly matched a known *M. balthica cox3* haplotype published in [29]. The Aytré population matched Nikula’s haplotype 37 (EF044099), belonging to the *M. balthica rubra* clade b1 (previously sampled from the Bay of Biscay); the Somme population matched haplotype 34 (EF044096) belonging to the same clade b1; the Gdansk population matched haplotype 16 (EF044078), belonging to the *M. balthica balthica* clade d2 (previously sampled from the Baltic Sea).

### Polymorphism Detection and Statistics

**Figure 4:**
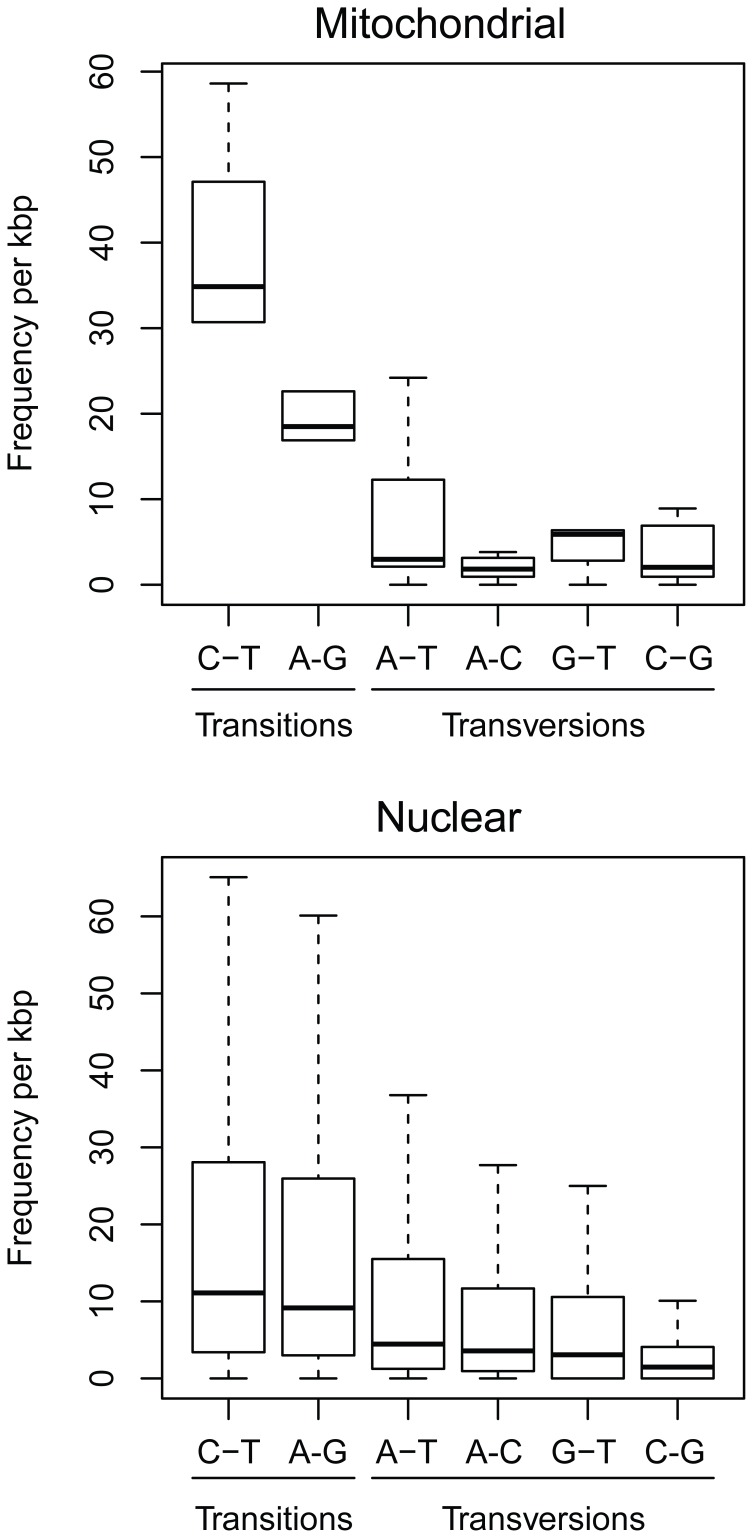
Frequency of transversions and transitions among high-quality mitochondrial and nuclear SNPs. Statistical outliers (values ≥1.5× the interquartile range) are omitted.

Of the 567,467 cleaned read sequences, 416,330 were mapped on reference contigs, and 54,606 putative SNPs were identified (1,103 and 53,503 for mitochondrial and nuclear contigs, respectively). Only SNPs characterized by a depth of coverage ≥10 in each population and a mean Minor Allele Frequency (MAF) ≥5% were considered in further analyses. Only 623 (mitochondrial) and 17,328 (nuclear) SNPs met these criteria. These high-quality SNPs were detected from 441 nuclear and 6 mitochondrial contigs. A high level of polymorphism was observed for the two compartments with 1 SNP every 19 bases for nuclear genes (i.e. 52.8 SNPs/kb) and 1 SNP every 14 bases for mitochondrial genes (i.e. 71.15 SNPs/kb). Transition:transversion ratios for these mutations were 1.22∶1 and 2.75∶1 for nuclear and mitochondrial contigs, respectively (Figure 4).

### Genetic Diversity and Population Differentiation

**Figure 5:**
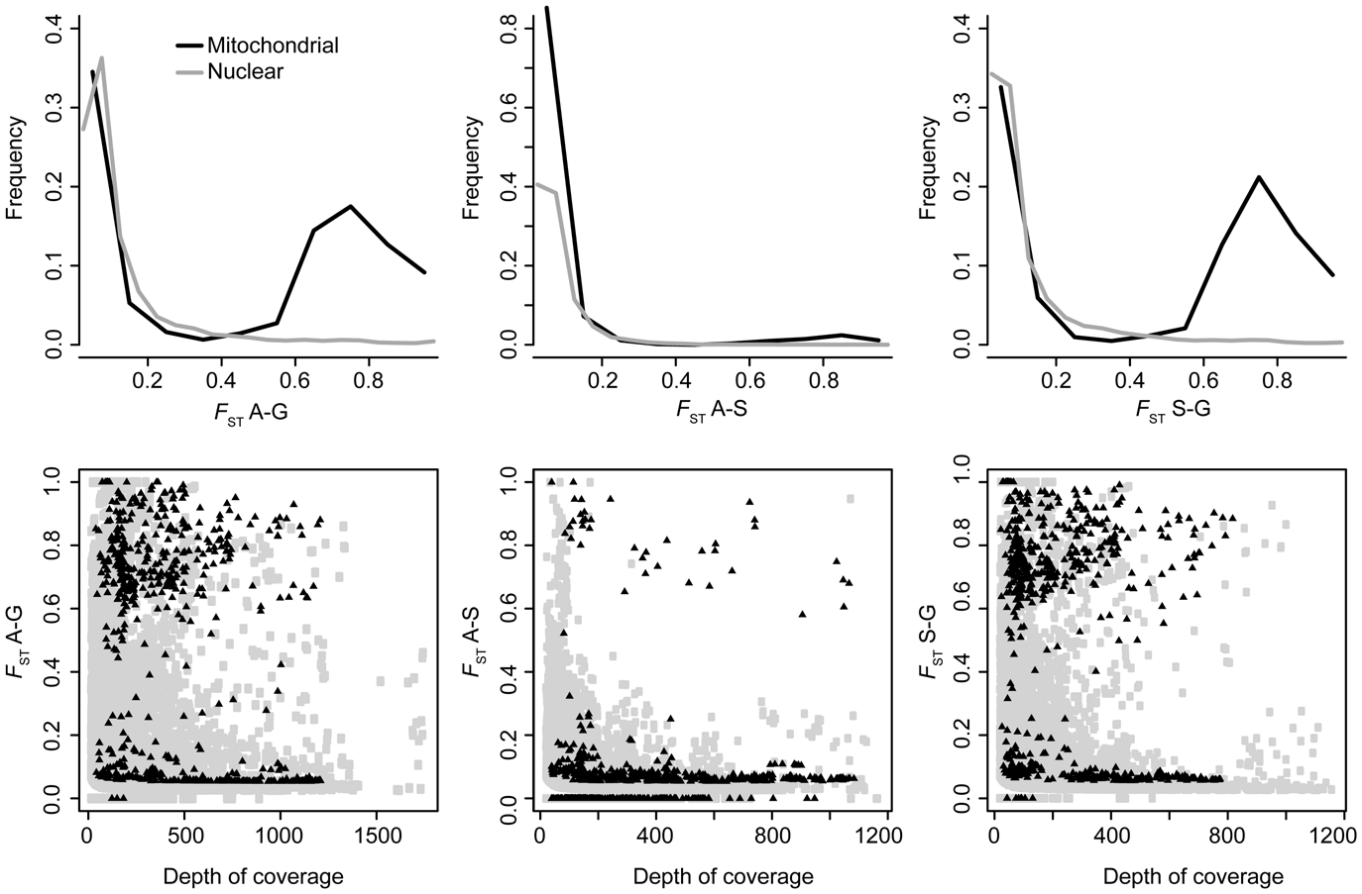
Pairwise 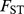 distributions. Top panel: frequency of 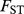 values for each pair of population. Bottom panel: relationship between depth of coverage and 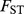 values. On both panels, nuclear and mitochondrial loci are represented in grey and black, respectively.

Average mitochondrial gene diversity (±SE) was 0.0065±0.0080, 0.0137±0.0083 and 0.0042±0.0049 for the Aytré, Gdansk and Somme populations respectively. Nuclear gene diversity was 0.0145±0.02, 0.0159±0.0189, and 0.0141±0.0192 for the Aytré, Gdansk and Somme samples. For nuclear loci, pairwise 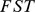 distributions were unimodal for all pairwise comparisons. Levels of genetic differentiation were low, with a median between 0.057 (Aytré-Somme) and 0.071 for Aytré-Gdansk. However, a few loci with high 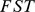 values were observed across all pairs (Figure 5). Indeed, maximum 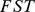 values were 1 for all pairs.

For mitochondrial loci, distributions of 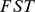 were bimodal especially in pairs with Gdansk, as expected knowing mitochondrial genealogies [27]–[29]. Medians of pairwise 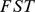 were 0.060, 0.665 and 0.644 for Aytré/Somme, Somme/Gdansk and Aytré/Gdansk respectively (Figure 5). Maximum 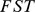 values reached 1 for all pairs of populations. We further characterized genetic variation at *cox1* and *cox3*, for comparison with previous studies [27]–[29]. Twelve diagnostic mutations were detected between the *M. balthica balthica* and *M. balthica rubra* lineages. Alleles corresponding to the *M. balthica rubra* lineage were nearly fixed within the Aytré and Somme populations (median frequency of 1.00), and rare with the Gdansk sample (allele frequency ranging from 0.005 to 0.104; median of 0.045). Twenty-two mutations separate the two lineages at *cox3* [29]. Except for 2 loci, alleles diagnostic of the *M. balthica rubra* lineage were nearly fixed within the Aytré and Somme populations (median frequencies of 1.00), and rare at Gdansk (frequencies ranging form 0.15 to 0.20; median of 0.16), except for one locus, which frequency reached 0.44 in the Gdansk sample.

### Outlier Detection

**Figure 6:**
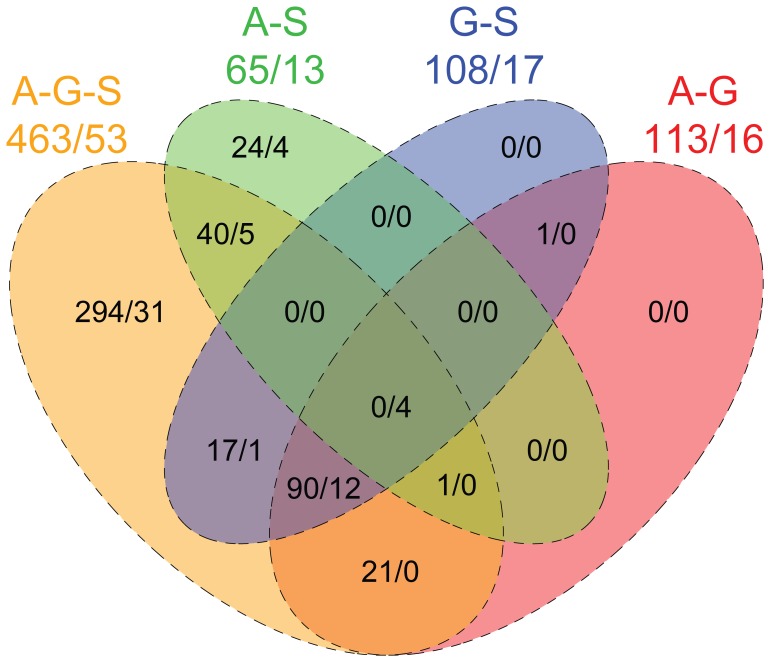
Venn diagram showing the intersect between the four outlier detection analyses. Each ellipse represents the consensus of three independent runs of BayeScan, for the three population pairs (A-G, A-S, G-S), and all populations (A-G-S). Numbers correspond to the total number of outlier SNPs (left of slash) and corresponding contigs (right of slash) for each analysis.

Out of 17,328 high-quality SNPs, 463 (2.7%) were identified as statistical outliers in our analyses involving all populations (i.e. loci in common between three independent BayeScan runs on the A-G-S set, in which 475, 477 and 478 outliers were detected). These outliers were clustered on 53 contigs, 28 of which could be identified using blast (Table S1). Eight contigs contained SNPs differentially fixed between population (i.e. 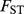  = 1), five of which returning a blast hit (Cyclophilin A, Myosinase I and II, Tropomyosine and vdg3). Three contigs were involved in ATP transport and synthesis. Six contigs (including the ATP synthase gamma subunit) contained SNPs corresponding to high 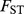 values for all three population pairs. One contig, corresponding to the Cyclin b gene (A_c226), was characterized by highly-differentiated SNPs for the Aytré-Somme population pair only. When population pairs were analyzed independently using BayeScan, 25 additional outliers were detected (24 from the A-S population pair, and 1 from the A-G and G-S pairs; Figure 6). These outliers were located on four contigs (two of which identified by blast as coding for the ATP synthase subunit alpha and Calmodulin) not involved in the outlier detection performed on the A-G-S set. 294 out of 463 outliers (63.5%) were only detected using the A-G-S set. The proportion of SNPs putatively under selection therefore varies between 1% (using population pairs only) and 2.8% (combining the results of all analysis sets). The relatively low proportion of overlap between analyses of the full set (A-G-S) and population pairs may be explained by the statistical power gained from using 30 individuals (A-G-S set) instead of 20 (population pair sets), by a higher false discovery rate in the A-G-S set [30], or a combination of both.

## Discussion

### Assembly and Polymorphism Detection

Three non-normalized cDNA libraries of pooled individuals allowed us to sequence 277 Mb of the transcriptome of *M. balthica*. The 454 technology is powerful in non model species for which little knowledge of the genome is available, and allowed us to produce contigs up to 4 kbp with a mean coverage close to 3x. Using larger contigs increased the number and accuracy of blast results and stabilized overall GC content, therefore decreasing the risk of analyzing contaminating DNA sequences. Indeed, almost half of the 1,714 reference contigs returned a blast hit with an e-value<1e−10 and 512 sequences were associated to a Gene Ontology term. These numbers are on par with previously-reported annotation success rates in mollusks [31]–[33], and reflect that the transcriptomics of this group are still poorly known. Results from blast searches did not provide any evidence of contamination by microalgae or bacteria. Most blast searches returned subject sequences corresponding to the cephalochordate *Branchiostoma floridae*, which complete genome was recently sequenced [34]. Additionally, top blast hits corresponded mainly to marine invertebrate species, including several bivalves.

### Technique Validation

The retrieval of documented mitochondrial haplotypes for the *cox1* and *cox3* genes allowed us to validate our methodology. For these two genes, haplotypes clustered in the expected mitochondrial clades (*M. balthica rubra* for Aytré and Somme populations and *M. balthica balthica* for Gdansk individuals), confirming that our quality filters seem to be appropriate. While comparing population consensuses to known *M. balthica* haplotypes, we also detected pseudogenes that would have otherwise been overlooked, as they were little divergent from orthologous sequences and contained few stop codons. This emphasizes the paramount importance of having a reference genome to which anonymous sequences can be compared, and warns that our sorting of mitochondrial and nuclear contigs is a mere hypothesis that remains to be fully tested. Nevertheless, putative mitochondrial contigs showed similar characteristics (relatively-high depth of coverage and high 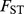 values). Interestingly, several contigs characterized as putative mitochondrial pseudogenes were detected as 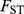 outliers, suggesting that either these pseudogenes were translocated into the nuclear genome after differentiation of mitochondrial loci (and the signature of population differentiation has not yet been diluted by drift), or that these pseudogenes are still under the influence of selection (e.g. [35]).

### Polyadenylation of Mitochondrial mRNAs

We used the Mint cDNA Synthesis Kit to produce our cDNA libraries. This kit uses adapters that anneal to the poly(A) of mRNA to initiate reverse transcription. The presence of poly(A) tails on mitochondrial mRNA molecules is therefore required for their processing into cDNA. The occurrence and role of polyadenylated mitochondrial mRNA is, however, highly variable among organisms [36], [37]. For example, this feature is completely absent in yeast [38], and triggers mRNA degradation in plants (e.g. [39]). Knowing whether mitochondrial mRNAs possess poly(A) tails is therefore paramount to properly annotating our contigs. Indeed, in the absence of poly(A) tails on mitochondrial mRNA, all contigs with high similarity with mt sequences must be nuclear pseudogenes (see [40] for a review of the prevalence of pseudogenes of mitochondrial origin in the nuclear genome). While data on mRNA polyadenylation is scant in non-model organisms [36], this mechanism has been hypothesized for some mitochondrial genes in the bellybutton nautilus [41] and the gastropod *Biomphalaria glabrata* [42], [43], based on the presence of incomplete stop codons. Focusing on the periwinkle *Littorina saxatilis*, Galindo *et al.* [44], [45] reported population differentiation data at NADH-like genes, based on 454 sequencing of pooled cDNA library produced using poly(A)+mRNA. While NADH genes are mitochondria-encoded, the authors recognized that their contigs could potentially be pseudogenes. The contigs that we selected as putative mitochondrial sequences have a homogeneously high depth of coverage (average >40 read/locus), and were characterized by similar differentiation profiles (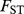 scan and Figure 5). Finally, we were able to match *cox1* and *cox3* sequences to known haplotypes produced by Sanger sequencing [27]–[29]. We therefore conclude that *M. balthica* is likely to feature polyadenylation of mitochondrial mRNAs in at least two genes.

### Extreme SNP Density

Simultaneous sequencing of individual pools is an economic alternative to the sequencing of individual genomes [46]. Although haplotype information is not retained if specimens are not individually tagged, it is possible to calculate reliable estimates of allele frequencies based on sampling effort, and therefore infer genetic diversity and population differentiation statistics [46], [47].

A major outcome of this study is the large SNP density recorded among three populations of *M. balthica* (1 SNP every 14 to 19 bp, for mitochondrial and nuclear loci, respectively). Sauvage *et al.* [48] recorded an average of one SNP every 60 nt in coding regions (1 SNP/40 nt in non-coding regions) in the Pacific oyster *Crassostrea gigas*, and concluded that the high prevalence of polymorphisms in this species was among the highest in the animal kingdom (other hyper-diverse taxa counting the nematode *Caenorhabditis remanei* [49]; the ascidian *Ciona savignyi* [50]; and *Drosophila* [51]). Comparisons of polymorphism density between *Macoma* and *Crassostrea* must, however, be done with care, as our study involved two sub-species characterized by three distinct mitochondrial lineages, while the 24 individuals used in Sauvage *et al.* where closely related (siblings and parent-offspring; [52]). These results therefore add to the existing view that bivalves might be champions of genetic diversity among animals (e.g. [48], [53]).

### Genome Scan for Population Differentiation and Cytonuclear Disequilibrium

2.7% of the tested nuclear loci were detected as outliers, based on a FDR of 5% and considering all three populations (A-G-S set). This number increased to 3.1% when the FDR was set to 10%. A review of 18 papers on AFLP-based genome scans showed substantial, but not extreme variation in the proportion of outliers, with a range from 0.4 to 24.5% and a mean of 8.5% [54]. A recent study of selection across the genome of *M. balthica* reported that 19% (using a coalescent approach, [55]) to 37% (using cline analysis, [56]) of 84 AFLP loci were under the influence of selection (only 3.6% of markers were detected by both methods) [57]. Genome scans based on SNPs revealed estimates of 7.5% in mice (*Mus musculus*, whole genome, [58]), 7–12% in the periwinkle (*Littorina saxatilis*, transcriptome, [45]), 1.4–3.6% in the Atlantic salmon (*Salmo salar*, expressed sequence tags, [59]), and 12% in the waterflea (*Daphnia magna*, expressed sequence tags, [60]). Additional estimates, within this range, were recently compiled by Orsini *et al.* [60]. The proportion of the transcriptome putatively under selection is therefore within the range of currently available estimates, although on its lower end.

Most recent studies aiming at detecting loci under divergent selection based on 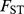 scans focus their interpretations of high population differentiation on “genetic-environment associations” that imply exogenous barriers to gene flow and local adaptation ([61] and references therein). Here, we detected loci associated with energetic metabolism, cellular respiration, and physiological stress (e.g. ATP synthesis genes, a heat shock protein, an immunosuppressant [cyclophilin A], and metalloproteases [myosinase I and II]), a result consistent with the fact that one population was sampled at the southern limit of the species range, and another was collected from heavily-polluted waters. It may therefore be tempting to conclude that a significant proportion of loci putatively under selection bear the molecular signature of local adaptation. Such loci are, however, expected to be rare at the scale of the entire genome and therefore very hard to pinpoint (e.g. [47], [62]). Genetic incompabilities such as Dobzhansky-Muller interactions, on the other hand, may form when previously-separated populations come into secondary contact, and generate endogenous barriers to gene flow. Endogenous barriers are at least as likely as exogenous ones, and may therefore explain a significant proportion of the highly-differentiated loci that we have detected [61].

An interesting outcome of our study was the high 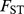 values recorded for the nuclear-encoded alpha, gamma and O subunits of the ATP synthase protein, an ATP/ADP transporter, and the second isoform of the nuclear NADH dehydrogenase, all of which being involved in electron transport and ATP synthesis at the mitochondrial membrane. The ATP synthase and NADH dehydrogenase genes contribute to the assembly of mitochondrial membrane proteins, which requires both nuclear- and mitochondrial-encoded units (e.g. [63]). As most SNPs detected for mitochondrial loci are characterized by high 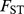 values between population pairs, the detection of nuclear genes involved in the electron transport system as 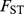 outliers strongly suggests cytonuclear incompatibilities (Dobzhansky-Muller incompatibilities between mitochondrial- and nuclear-encoded proteins), an endogenous barrier to gene flow symptomatic of hybridizing lineages (e.g. [64]–[70]). The hypothesis that genetic incompatibilities contribute to population differentiation in *M. balthica* is consistent with the complex colonization history of this species in the Atlantic [16], [29], [71]–[73]. Indeed, the last few million years have seen multiple colonization of Pacific *M. balthica* into Atlantic waters through the Bering Strait, leading to secondary contact between divergent lineages and extensive hybridization.

While describing the genetic structure of *M. balthica* populations in the Baltic, White and Barents Seas, Strelkov *et al.* [73] and Nikula *et al.* [72] statistically tested for evidence of cytonuclear disequilibrium among mitochondrial (*cox3*) and nuclear (10 allozyme loci) markers, but did not detect any. This genome-wide scan for genetic differentiation therefore provides the first signal of cytonuclear disequilibrium in *M. balthica*. As nuclear-encoded ATP synthase subunits proved to be outliers, it would be interesting to investigate further the rate of molecular evolution of mitochondrial-encoded subunits [69]. Unfortunately, these genes (*atp6* and *atp8*) could not be detected among the 1,714 quality-filtered contigs, or among the raw 58,304 contigs produced by our MIRA assembly. Future research efforts will therefore focus on further characterizing the extent of cytonuclear disequilibrium among hybrid populations of *M. balthica*, and contrast it with background levels from natural populations (e.g. [74]).

### Conclusions and Future Research Efforts

*M. balthica* is a particularly interesting model system for the study of hybridization in postglacial marine environments [29], [73], [75], and genetic information is now accumulating rapidly for this species, as two mitochondrial genes (*cox1* and *cox3*) [27]–[29], [72], [73], nine nuclear microsatellite loci [16], [76], 17 allozyme loci [71], [77], and 84 AFLP loci [57] were produced to date. The avalanche of data produced with the 454 platform allowed us to scan for nuclear and mitochondrial genes putatively under the influence of selection, detect about 17K SNPs, estimate their density across the genome, and pave the way to high-throughput population genetics for this species. Indeed, we were able, based on this preliminary scan, to identify 384 SNP markers that will be tested in mass genotyping across the full biogeographic range of *M. balthica* in the NE Atlantic, spanning two zones of strong population differentiation. SNPs diagnostic of population differentiation will be statistically tested for selection (e.g. [55], [78]). Based on the preliminary annotation presented here, and future transcriptome sequencing efforts, outlier SNPs will further inform us on which genes are involved in the establishment and maintenance of hybrid zones of different age and origin, and their dynamics in the face of rapid climate change [6], [7].

## Materials and Methods

### Ethics Statement

No specific permits were required for the described field studies in Aytré, France and in the Gulf of Gdansk, Poland. Sampling locations were not private nor protected. Collection of *M. balthica* in the Somme Nature Reserve, France, was approved by Mr. Patrick Triplet from the Reserve administration. Field studies did not involve endangered or protected species.

### Sampling and Sequencing of cDNA Libraries

**Figure 7:**
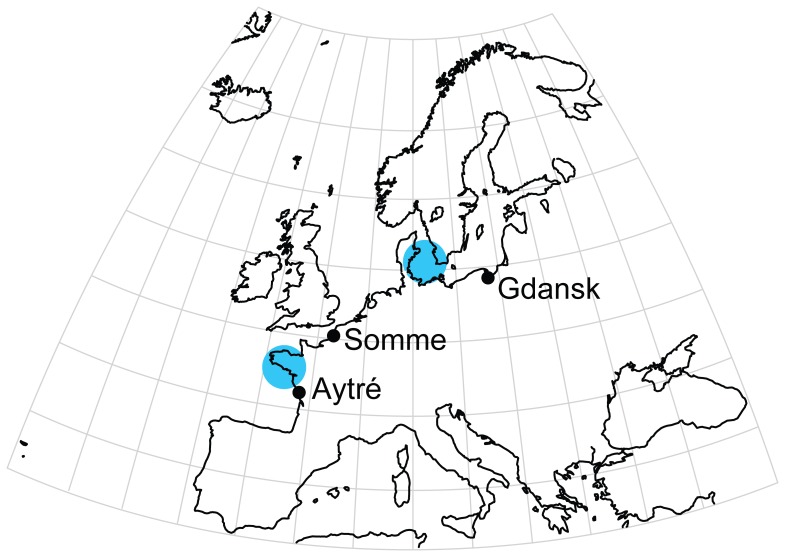
Sampling locations of the three population pools used for SNP discovery in *Macoma balthica*. Orthogonal projection with 10 degree grid. The positions of contact zones among differentiated mitochondrial lineages are indicated as blue circles (after [16], [72]).

To maximize the likelihood to detect high polymorphism levels and develop SNP markers relevant to the study of hybrid zones and local adaptation, we chose to sample three geographically disjunct populations (Figure 7). The population of Aytré (Bay of Biscay, France; 46.13N 1.13W) is located at the southern limit of the species distributional range, is adjacent to a putative hybrid zone in southern Brittany [16], and is subjected to warming surface waters [11]. The population of Gdansk (Baltic Sea, Poland; 54.21N 18.68E) was sampled near the Vistula River. This population is located eastward of a well-documented hybrid zone [29], [72]. The population of the Somme Bay (English Channel, France; 50.21N, 1.62W) is located in a nature reserve, and at the heart of the biogeographic range of *M. balthica*. Collections occurred in January 2008, and ten individuals were sampled per site. Whole individuals were preserved immediately after collection in RNAlater (Sigma) to stabilize cellular RNA. Total RNA was extracted with TRIzol (Invitrogen), quantified with a NanoDrop ND-1000 spectrophotometer (Thermo Fisher Scientific) and using 1% agarose gels, and diluted to 400 ng/*μ*l. Pooling individuals into a single sequencing run allows the estimation of allele frequencies and population differentiation statistics at a lower cost than with individual tagging [46], [79], [80]. The RNA extracted from 10 individuals, for each population, was therefore pooled, and three cDNA libraries were constructed using the Mint cDNA Synthesis kit (Evrogen). cDNA quality was checked on a 1.2% agarose gel in 1X TAE buffer before purification with the NucleoSpin Extract II Kit (MACHEREY-NAGEL GmbH & Co.). Libraries were not normalized in order to maximize depth of coverage. Twenty *μ*g of each cDNA library were sent to Beckman Coulter Genomics (Grenoble, France) to be analyzed on a single Roche 454 GS-FLX Titanium sequencing run. Each cDNA library was tagged prior to sequencing in order to separate reads from different populations after the sequencing step.

### Assembly

Primer sequences, poly(A) tails and reads produced from ribosomal DNA template were removed with SeqClean [81]. Cleaned reads were deposited to the NCBI Short Read Archive (Submission # SRA052276.1, Accession # SRX145744-6, [82]). Each of the three sets of sequences was individually assembled with MIRA v. 3.0.0 [83] with the following parameters: 454 sequencing technology; accurate, *de novo* EST assembly type). Resulting contigs were then pooled. Because the aim of the study was not to sequence the entire transcriptome but rather to detect polymorphic sites from high-quality sequences, we restricted our analyses to contigs with average quality â≥42, built de novo from â≥10 reads, and longer than 400 bp. Duplicated contigs were removed after detection using clustering (CD-HIT-EST with default parameters; [84]), reciprocal blast, and custom perl scripts (available upon request). Singletons and contigs built in repetitive regions (“rep”, as detected by MIRA) were also removed.

### Contig Identification and Functional Annotation

Automated local blastx search (NCBI “nr” database with e-value of 1e−10 and HSP length cut-off of 100) and gene annotation (using default parameters and evidence code weights) were performed with Blast2GO v. 2.6.0 (database version b2g_jun11) [85]–[88]. Mitochondrial sequences were identified using local blast on the complete mitochondrial genome of *Sinonovacula constricta* (EU880278.1). To detect putative pseudogenes, the selected contigs were then further characterized by the prevalence of stop codons in the six reading frames using NCBI ORF Finder [89].

### Read Mapping and SNP Discovery

In this communication, coverage is referred as the number of time a particular reference DNA fragment was sequenced; depth of coverage is defined as the number of reads providing information about a particular base [90]. Contigs were used as reference genome for SNP discovery (1,714 contigs, equivalent to 1.96 Mb). To guide the mapping step, a hash index for those contigs was built in SMALT v. 0.5.8 (Wellcome Trust Sanger Institute 2010, 2011 Genome Research Limited [91]) using a word length of 13 (k-mer) and a sampling step size of 2, as recommended in the SMALT manual for Roche 454 sequence data. Read sequences and quality scores were combined into a fastq file using the Galaxy pipeline [92]–[95], and mapped onto the 1,714 reference contigs using SMALT. The resulting SAM files were filtered in SAMtools v. 0.1.17 [96] to retain alignments with a minimum quality score of 20, and individuals base calls with a minimum quality score of 20. This last threshold was found to be generally sufficient for the detection of high-quality SNPs from PoolSeq data on the Illumina platform [97]. Only SNPs with a minor allele frequency (averaged over all populations) â≥5% and a depth of coverage â≥10 reads for each population were considered. Mitochondrial and nuclear SNPs were treated separately. Data were manipulated using custom R scripts [98]–[100].

### Population Genetic Summary Statistics and Outlier Detection

Allele frequencies were computed for each population. Because of the atypical sampling properties due to pooling of individuals, pairwise 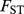 and expected heterozygosity were computed as in [47]. Gene diversity was computed as expected heterozygosity averaged over the total number of sites with coverage above 10. Outlier detection was performed on nuclear SNPs using the bayesian method implemented in BayeScan v2.0 [101]. In a recent benchmark of 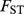 outlier tests for SNPs, BayeScan was found to provide high power and low rates of type I errors [102]. To evaluate the impact of the hierarchical genetic structure between the three populations [30], we performed a total of four analysis sets: one including information from all three populations (A-G-S), and three corresponding to the different population pairs (A-G, A-S, G-S). Short pilot runs (20 pilot runs of length 5,000) were used to estimate model parameters. Sample size was then set to 5,000 and the thinning interval to 10, for a total MCMC chain length of 100,000 steps, of which the first 50,000 were discarded (burnin). Given the large number of SNPs to test, prior odds were set to 1,000 (BayeScan manual). Outlier detection was done with a false discovery rate (FDR) set to 5%, corresponding to a Bayes Factor (BF)>10, suggesting strong evidence for selection according to Jeffreys’ scale [103]. Three independent runs were performed to check for result consistency within each analysis set. Convergence was assessed by checking that no temporal trends in log-likelihood plots were visible.

## Supporting Information

Table S1
**Description of nuclear contigs with outlier loci.** Contigs identified as â “hypothetical protein” using blast were reported as “non-annotated” (na). Accession numbers and E-values correspond to the blast top hit. Information on SNPs that are fixed in some populations (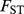  = 1) is bolded.(XLS)
