# Correction: SNP Detection from *De Novo* Transcriptome Sequencing in the Bivalve *Macoma balthica*: Marker Development for Evolutionary Studies

**DOI:** 10.1371/annotation/40a74b75-fdcc-4c15-9ddb-cf6ac40464f7

**Published:** 2013-09-09

**Authors:** Eric Pante, Audrey Rohfritsch, Vanessa Becquet, Khalid Belkhir, Nicolas Bierne, Pascale Garcia

Institutional affiliations 2 and 3 for authors Khalid Belkhir and Nicolas Bierne are incorrect. Affiliations 2 and 3 should be: UMR 5554, ISEM Université Montpellier II.

In addition, the term "FST" was incorrectly typeset multiple times in the "Genetic Diversity and Population Differentiation" section of the Results. The correct typesetting of this term is F_ST_.

